# The temperature sensitivity of soil organic carbon decomposition is not related to labile and recalcitrant carbon

**DOI:** 10.1371/journal.pone.0186675

**Published:** 2017-11-02

**Authors:** Jie Tang, Hao Cheng, Changming Fang

**Affiliations:** Ministry of Education Key Laboratory for Biodiversity and Ecological Engineering, The Institute of Biodiversity Science, Fudan University, Shanghai, China; Tennessee State University, UNITED STATES

## Abstract

The response of resistant soil organic matter to temperature change is crucial for predicting climate change impacts on C cycling in terrestrial ecosystems. However, the response of the decomposition of different soil organic carbon (SOC) fractions to temperature is still under debate. To investigate whether the labile and resistant SOC components have different temperature sensitivities, soil samples were collected from three forest and two grass land sites, along with a gradient of latitude from 18°40’to 43°17’N and elevation from 600 to 3510 m across China, and were incubated under changing temperature (from 12 to 32 ^o^C) for at least 260 days. Soil respiration rates were positively related to the content of soil organic carbon and soil microbial carbon. The temperature sensitivity of soil respiration, presented as *Q*_10_ value, varies from 1.93 ± 0.15 to 2.60 ± 0.21. During the incubation, there were no significant differences between the *Q*_10_ values of soil samples from different layers of the same site, nor a clear pattern of *Q*_10_ values along with the gradient of latitude. The result of this study does not support current opinion that resistant soil carbon decomposition is more sensitive to temperature change than labile soil carbon.

## Introduction

Terrestrial ecosystems play an invaluable role in regulating global carbon cycle. The amount of CO_2_ emitted from the soil is approximate to or exceeds the global net primary production [1], and several times higher than the amount of CO_2_ emitted by the consumption of fossil fuel [2]. The temperature dependency of SOC decomposition and its possible future variation are major concerns regarding the interaction between global climate change and terrestrial ecosystems [3].

The temperature sensitivity of different SOC fractions is an element of uncertainty, and thus is a highly debated topic with regard to global climate change [4]. Currently, there are no widely accepted theories or hypothesis on the main mechanism [5]. The opinion that the decomposition of labile carbon is more sensitive to temperature change than the resistant carbon is mainly reported in earlier studies [6,7]. Model analysis had showed that soil organic matter turnover coupled with a model of net primary production response to temperature [7]. Many studies had reported that the labile and resistant SOC components had similar sensitivities to temperature change [8–10]. Several other studies again conform to the notion of resistant carbon fractions being more sensitive to temperature change than labile carbon fractions [11–15]. Otherwise, temperature sensitivity increased with increasing soil depth and decreasing SOM quality through a 495—day laboratory incubation [16]. However, it is still unclear whether the decomposition of different SOC components responds differently to temperature variation.

The temperature sensitivity of SOC decomposition is the response of SOC decomposition rate to soil temperature. As a chemical or microbial mediated process, the SOC decomposition could be described by chemical reaction kinetics equation, so that the temperature effect on the SOC decomposition is usually quantified by the temperature coefficient of the decomposition reaction rate. The temperature coefficient, used to characterize the temperature sensitivity, is commonly referred to as *Q*_10_ value, presenting the respiration rate differs for a temperature interval of 10°C. Changes in observed *Q*_10_ values for soil respiration fluctuated worldwide between 1.0 and 3.5, with an average value of around 2.5, both in the field (including plant root respiration) and laboratory incubation with or without root respiration [17]. *Q*_10_ value has significant spatial and temporal changes [18,19], leading to a controversy in the prediction of future response of SOC in terrestrial ecosystems to global climate change.

The *Q*_10_ values are usually calculated by different soil respiration models, with the soil respiration or SOC decomposition rates measured under either field observation or laboratory incubation. Both the soil respiration models and soil respiration rates measurement methods may lead bias on the *Q*_10_ values. Most soil respiration models, from the first-order reaction kinetics to the Arrhenius equation and so on, can simulate the relationship between the soil respiration rate and the temperature correctly. However, there are significant differences in the *Q*_*10*_ values derived from the different respiration models, because of different chemical kinetics or biological theories. The soil respiration or SOC decomposition measurements can also lead different results. The field observation have too many uncontrollable factors other than temperature, such as water condition, roots biomass, carbon input and etc., which can affect soil respiration and confuse *Q*_10_ calculation [19,20]. In laboratory incubation of soils, a commonly used method is to divide soil samples into several groups and incubate separately at different but constant temperatures, and the *Q*_10_ value was estimated by using respiration rates measured at different temperatures [21], the lost carbon mass during a certain time [18], or the time required for a certain amount carbon respired [22]. However, it was pointed out that former two methods may underestimate while the last one may overestimate *Q*_10_ value, because of the faster depletion of labile C and decrease in soil microbes along with the incubation under relatively higher temperature. Furthermore, incubating soils at different fixed temperatures caused changes in microbial composition, resulting in different temperature sensitivities during the incubation [18]. Because of the impacts of experimental methods on estimated *Q*_10_ value, the difference of *Q*_10_ values of different SOC components remains unclear.

In this study, for obtaining precisely soil respiration rates of incubation condition, a fast-incubation method is designed, which will minimize the disruption of affecting factors other than temperature [8]. This method was also repeated, and found no significant impact by experimental procedures of this method on estimated *Q*_10_ value of soil respiration [23]. And, to make results comparable between different samples, we used the first-order reaction kinetics equation to estimate the *Q*_*10*_ values of different sample, which provided a stable *Q*_*10*_ value for a sample during the whole incubation temperature region.

To detect the differences in the temperature sensitivity between different SOC fraction, soils developed under different ecosystems across climate zones in China were collected and incubated in the laboratory under changing temperature, measured soil respiration rate were used to estimate the temperature sensitivity of soil heterotrophic respiration or SOC decomposition. Our objectives were to: analyze the relationship between soil respiration rate and temperature from different soil backgrounds; investigate the variations in temperature sensitivity of soil respiration under different conditions; estimate the *Q*_10_ values of soil resistant and labile C components in different soils and assess its implication in current global climate change.

## Materials and methods

### Sites and soil sampling

Five sites across China, along with a gradient of latitude from 18°40′ to 43°17′N and elevation from 600 to 3510 m, were selected. Of which three were forests: a tropical rain forest in Hainan Province (in the observatory research station of Institute of Subtropical Agriculture, Chinese Academy of Sciences, and authorized by the institute), a subtropical evergreen forest in Fujian Province, (in the area of the Nature Reserve of Wuyi Moutain, and authorized by the nature reserve administration) and a temperate deciduous forest in Changbai mountain (in the observatory research station of Institute of Applied Ecology, Chinese Academy of Sciences, and authorized by the institute); and two were pasture: a semiarid steppe grassland in the Inner Mongolia (in the area of Xilin Gol League, and authorized by the Village government), and a cold temperate meadow in the northern edge of Tibetan Plateau in Gansu Province (in the area of the Nature Reserve of Gahai-Zecha, and authorized by the nature reserve administration). Climate of these sites varies from tropical monsoon climate to plateau continental monsoon climate, with a MAT gradient 20 to 2.6°C. Detailed information of each site was listed in Tables 1 and 2.

**Table 1:** Study sites characteristics.

Site	ID	Location	Climate region	Altitude(m)	Vegetation type	Dominant species	MAP* (mm)	MAT** (°C)	Soil taxomony
**Tropical rainforest**	RF	N 18°40’	Tropical monsoon climate	600	Forest	*Mallot us hookerianus*	2650	20	ferralsol
		E 108°49’				*Gi ronniera subaequalis*			
**Evergreen forest**	EF	N 27°30’	Subtropical monsoon climate	500	Forest	*Castanopsis eyrei* Tutch	2400	17	ferralsol
		E 117°35’				*Cyclobalanopsis multinervis*			
**Deciduous forest**	DF	N 42°24’	Temperate continental mountain climate	740	Forest	*Pinus koraiensis* Sieb. et Zucc	1100	4.6	alfisol
		E 128°28’							
**Temperate meadow**	TM	N 34°15’	Plateau continental monsoon climate	3510	Grass land	*Kobresia myosuroides (Villrs)* Fiori	780	1	aquisols
		E 102°21’							
**Semi-arid grassland**	SG	N 43°17’	Semi–arid temperate grassy climate	980	Grass land	*Leymus chinensis*	350	2.4	primarosols
		E 116°02’				*Stipa krylovii*			

**MAT: mean annual temperature

*MAP: mean annual precipitation

**Table 2:** Chemical characteristics of soils from different sites.

	Surface soil					Subsurface soil	
	Semi-arid grassland	Temperate meadow	Deciduous forest	Evergreen forest	Tropical rainforest	Semi-arid grassland	Deciduous forest
**pH(H**_**2**_**O)**	8.1	6.3	5.5	5.2	5.5	8.2	5.1
**WHC(%)**	19.4	30.5	33.0	26.0	20.1	18.3	42.5
**NO**_**3**_^**—**^**N**	3.97	7.38	6.76	5.06	9.22	2.45	5.15
**NH**_**4**_^**+**^**-N**	0.40	0.56	2.66	0.80	3.47	0.35	0.81
**TN**	1.27	5.82	2.94	1.79	0.60	0.18	0.56
**SOC**	9.55	99.10	77.00	33.40	7.41	1.25	7.73
**K**_**2**_**SO**_**4**_**-C**	0.06	0.17	0.05	0.10	0.07	0.02	0.06
**MBC**	0.07	0.16	0.18	0.09	0.02	0.02	0.03

WHC: water holding capacity; TN, total nitrogen; SOC, soil organic carbon; MBC, microbial biomass carbon; TC, total carbon.

Units are mg kg^-1^ for NO_3_-N and NH_4_^+^-N, and mg g^-1^ for TN, SOC, K_2_SO_4_-C and MBC.

At each site, soil samples were collected from three locations approximately 10 meters apart from each other. After cutting-off above ground plants and removing the surface litter, pits were dug to a depth of 60 cm at maximum, dependent on the actual depth of soil at individual site. Soil samples were collected from the surface soil layer (0–10 cm) (all five sites) and subsoil layer (20–30 or 50–60 cm) (only deciduous forest and semiarid steppe grassland sites). After roots and rocks removed, the samples were sieved through a 2mm mesh, and then composited by soil layer and site. Fresh soil samples were then brought back to laboratory as soon as possible.

### Soil analysis

Soil pH value was measured in water extract (soil:water = 1:2.5) using a glass electrode. Water holding capacity (WHC) was determined by drying soils at 105°C for 48 h, which had been water saturated and allowed to drain over 2 h. For determining NH_4_^+^-N and NO_3_^—^N concentrations, the soil was extracted with KCl solution (2 M) and filtered, and then was measured using a colorimetric assay at 625 nm and 220 nm. Total nitrogen (TN) in the soil was measured by a NC analyzer (FlashEA 1112 Series, Italy) with combustion at 625°C. The measurement of total organic carbon (TOC) was done by using a TOC analyzer. Soil microbial biomass carbon (MBC) was determined by the fumigation-extraction method. K_2_SO_4_—C was extracted with K_2_SO_4_ solution (0.5 M) and filtered, determined by using a TOC analyzer [8].

### Laboratory incubation and respiration measurements

A programmable incubation system, modified from previous study [8], was developed. Sixteen stainless steel chambers, of a height of *ca* 21 cm with an inner diameter of *ca* 11 cm, were aligned in 4×4 with a minimal distance of 4 cm between chambers. Chambers were ironed, at the position 6 cm to the upper end, to a stainless steel plate. The lower two thirds of chambers, i.e. the part beneath the stainless steel plate, were submerged in water. The upper end of chambers was closed by air-tight caps with a perspex window and a small outlet. The small outlet was always closed except the time of taking gas samples when it was connected air-tightly to a syringe. The lower part of the outlet, i.e. the part inside the chamber, was a needle with an open in chamber center at *ca* 4 cm under the perspex window.

An air distribution system passed fresh air uniformly and horizontally through each chamber, via a side inlet and outlet at the position of *ca* 3 cm from the upper end of chambers, to simulate the field condition of air flowing above soil surface. Fresh air was drawn from the top of the building, passed through a water bath, and stabilized in a *ca* 250 liter buffer. Air was then kept flowing continuously through each chamber, at a rate of about 50 ml min^-1^ per chamber, except the period of measuring respiration when the chamber was completely closed.

A water circulation system ensured the spatial variation in temperature, monitored by 5 sensors, within ± 0.2°C. After a pre-experiment, the incubation temperature was set to change between 12 and 32°C, starting from the lowest, continuously increasing to the highest and then decreasing back to the lowest with a change step of 4°C. Two hours were allowed for incubation system to reach a new temperature, i.e. 1°C per half hour, in order to avoid a quick heating or cooling of soil samples. The system was then held at the desired temperature for 1 hour before starting respiration measurement.

For measuring soil respiration, a first gas sample of 20 ml was taken from each chamber, the chamber was then closed immediately, and a second gas sample of 20 ml was taken 20 minutes to 1 hour after the chamber was closed, depending on the change in CO_2_ concentration in gas samples at different temperatures. The CO_2_ concentration in gas samples was measured, using an infra-red gas analyzer (Licor 7000, Licor Company, USA). Soil respiration rate was determined by the difference in CO_2_ concentration between the two gas samples, the time interval of closing chamber, the actual volume of head space of each chamber and dry soil weight.

Four replicates of each soil sample, by site and soil layer, were incubated in the laboratory for various lengths of time period, the shortest over 250 days and the longest over 500 days. Each replicate of soil samples, about 600 g to 1 kg fresh soil depending on bulk density and adjusted to about 60% WHC, was contained in a specially made stainless steel container. The container was *ca* 15 cm in height with an outside diameter of *ca* 10.8 cm. Each container has 4 sub-containers of quarter-circular cylinder, also made of stainless steel, to fully match the inner space of the container. Soils were packed into sub-container to their original bulk densities and have a similar volume among different containers. As the incubation system can only hold 16 soil containers at a time, soil containers were incubated with the system in turn. In other times, soil containers were held in a green house, with direct sun light being shaded and moisture checked and adjusted regularly. The green house temperature was not controlled.

### Q_10_ calculation

The relationship between soil respiration and temperature has been commonly described by a first-order exponential equation:

$$R_{T}=R_{0}\cdot e^{\beta T}$$ (1)

where *R*_T_ is soil respiration rate at temperature *T* (°C), *R*_0_ is a fitting parameter, referring to as the soil respiration rate at 0°C, and *β* is also a fitting parameter, commonly referred to as the temperature coefficient.

The *Q*_10_ value of soil respiration is conceptually defined as:

$$Q_{10}=\frac{R_{T+10}}{R_{T}}$$ (2)

where *R*_T_ and *R*_T+10_ are respiration rates at temperatures of *T* and *T*+10, respectively [21]. For above first-order exponential equation, the *Q*_10_ value can be calculated as:

$$Q_{10}=\frac{R_{0}\cdot e^{\beta (T+10)}}{R_{0}\cdot e^{\beta T}}=e^{10\beta }$$ (3)

Significant differences among the means were determined by ANOVA tests at the 5% level. Data were performed using the SPSS 19.0 software package.

## Results

### Soil respiration

**Fig 1:**
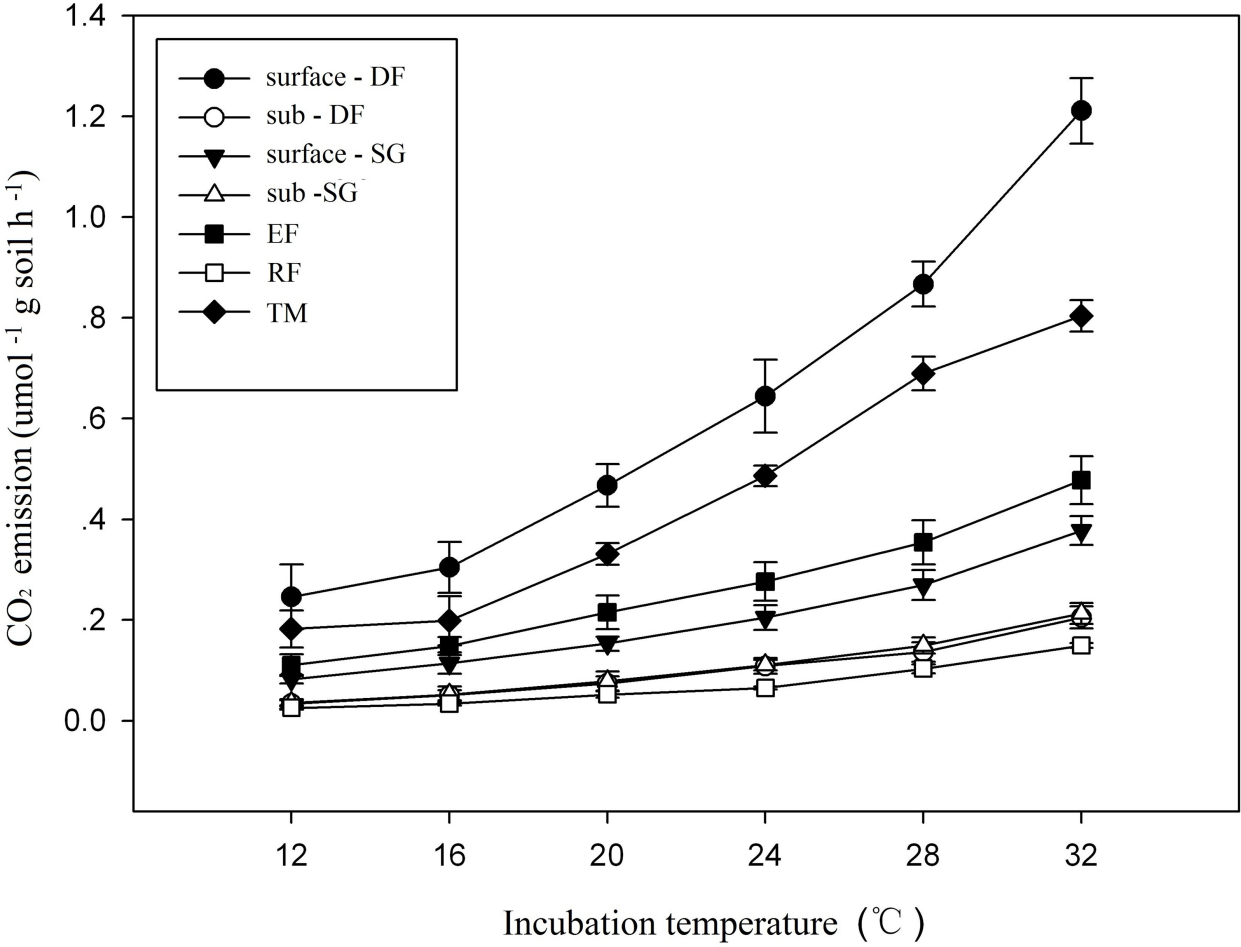
Relationship between SOC decomposition rates and incubating temperatures for soil samples from different sites. (DF: deciduous forest; TM: temperate meadow; RF: tropical rainforest; EF: evergreen forest; SG: semi-arid grassland).

Soil respiration rates, expressed on the base of dry soil weight, increased exponentially with incubation temperature between 12 to 32°C in all soil samples (Fig 1). At the beginning of incubation, respiration rates at 20°C were 0.15, 0.33, 0.05, 0.22 and 0.44 μmol CO_2_ g dry soil^-1^ h^-1^ for the surface soil samples from semi-arid grassland, temperate meadow, tropical rainforest, evergreen forest and deciduous forest, respectively. In subsoil soil samples, respiration rates were significantly lower than that of surface soil, e.g. only 0.1 and 0.07 μmol CO_2_ g soil^-1^ h^-1^ for subsoil from semi-arid grassland and deciduous forest. Respiration rates across all soil samples had a significantly positive relationship with the content of SOC and MBC (p < 0.05).

**Fig 2:**
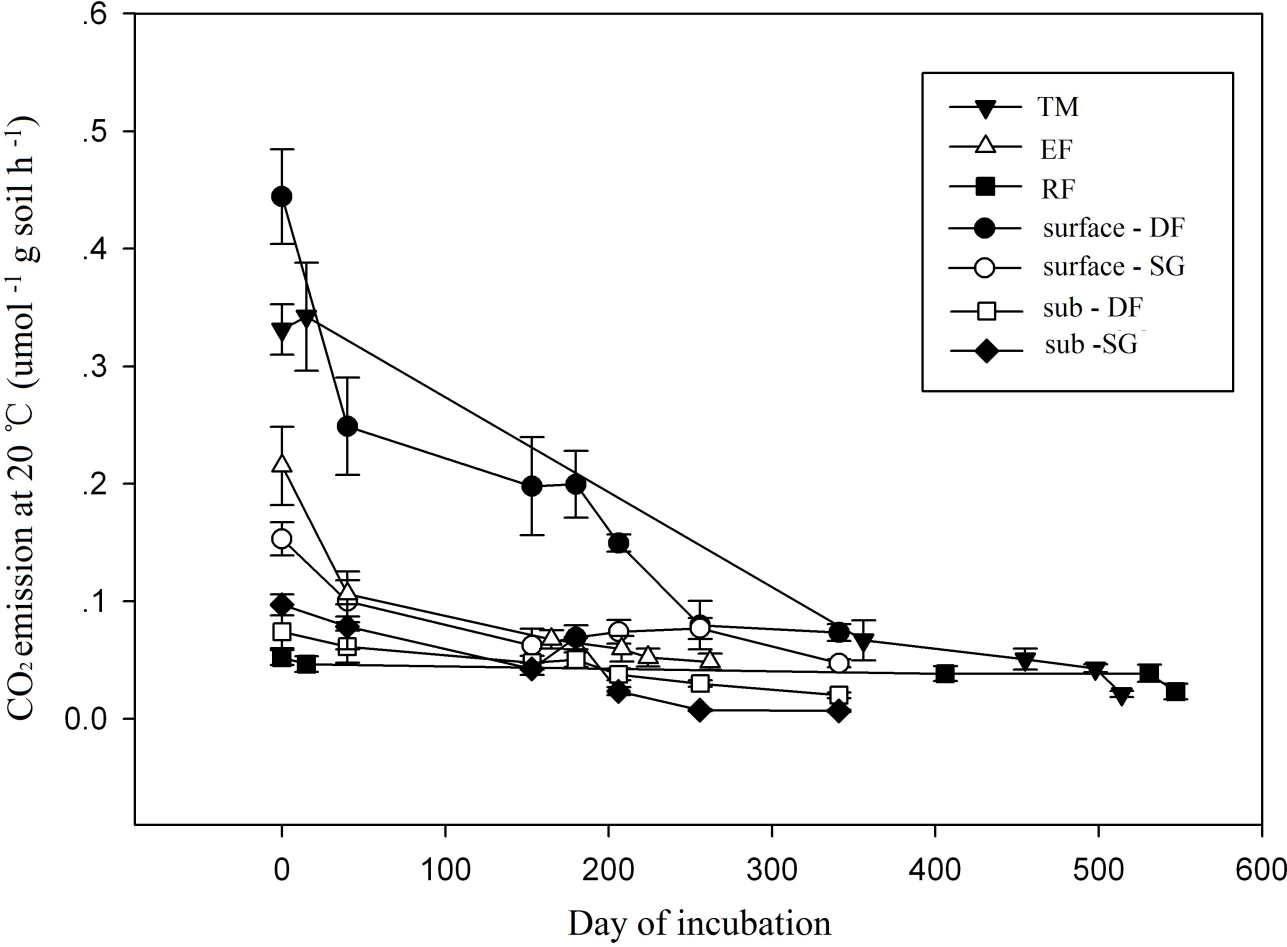
SOC decomposition rates (at 20 ^o^C) declined over incubation times. (DF: deciduous forest; TM: temperate meadow; RF: tropical rainforest; EF: evergreen forest; SG: semi-arid grassland).

Along with the progress of incubation, soil respiration rates at 20°C declined with time in all soil samples, and rapid decline mainly occurred within the first 100 days (Fig 2). At the end of incubation, respiration rates were only 31.0%, 16.0%, 44.6%, 22.5% and 16.5% of the initial values in the surface samples from semi-arid grassland, temperate meadow, tropical rainforest, evergreen forest and deciduous forest, respectively. The corresponding values were 7.1% and 27.1% for subsoil samples from semi-arid grassland and deciduous forest.

### Effects of incubation time on Q_10_ value

By fitting respiration rates with Eq 1, estimated *Q*_10_ values of all soils varied from 1.93 ± 0.15 to 2.60 ± 0.21 (Table 3). Temperate meadow soil had the lowest and semi-arid grassland soil had the highest *Q*_10_ value.

**Table 3:** Variation in *Q*_*10*_ values (mean ± SD) along with incubation.

**Surface-DF**		**Sub-DF**		**TM**		**RF**	
day	Q_10_	day	Q_10_	day	Q_10_	day	Q_10_
0	2.25±0.12	0	2.36±0.21	0	2.14±0.16	0	2.52±0.14
40	2.16±0.23	40	2.33±0.17	15	2.10±0.11	15	2.42±0.20
153	2.23±0.21	153	2.29±0.18	356	2.31±0.24	306	2.47±0.25
180	2.20±0.31	180	2.19±0.25	455	1.84±0.13	431	2.38±0.33
206	2.24±0.16	206	2.20±0.26	498	1.93±0.15	547	2.35±0.19
256	2.50±0.23	256	2.33±0.23	514	2.10±0.26	-	-
341	2.46±0.16	341	2.35±0.13	-	-	-	-
**EF**		**Surface-SG**		**Sub-SG**		-	
day	Q_10_	day	Q_10_	day	Q_10_		
0	2.02±0.13	0	2.47±0.20	0	2.36±0.19		
41	2.24±0.32	60	2.48±0.21	60	2.24±0.23		
165	2.06±0.23	140	2.59±0.36	140	2.59±0.36		
208	2.33±0.23	166	2.50±0.17	166	2.60±0.21		
224	2.19±0.15	216	2.54±0.14	216	2.53±0.23		
262	2.23±0.18	301	2.56±0.12	301	2.55±0.22		

DF: deciduous forest; TM: temperate meadow; RF: tropical rainforest; EF: evergreen forest; SG: semi-arid grassland

**Fig 3:**
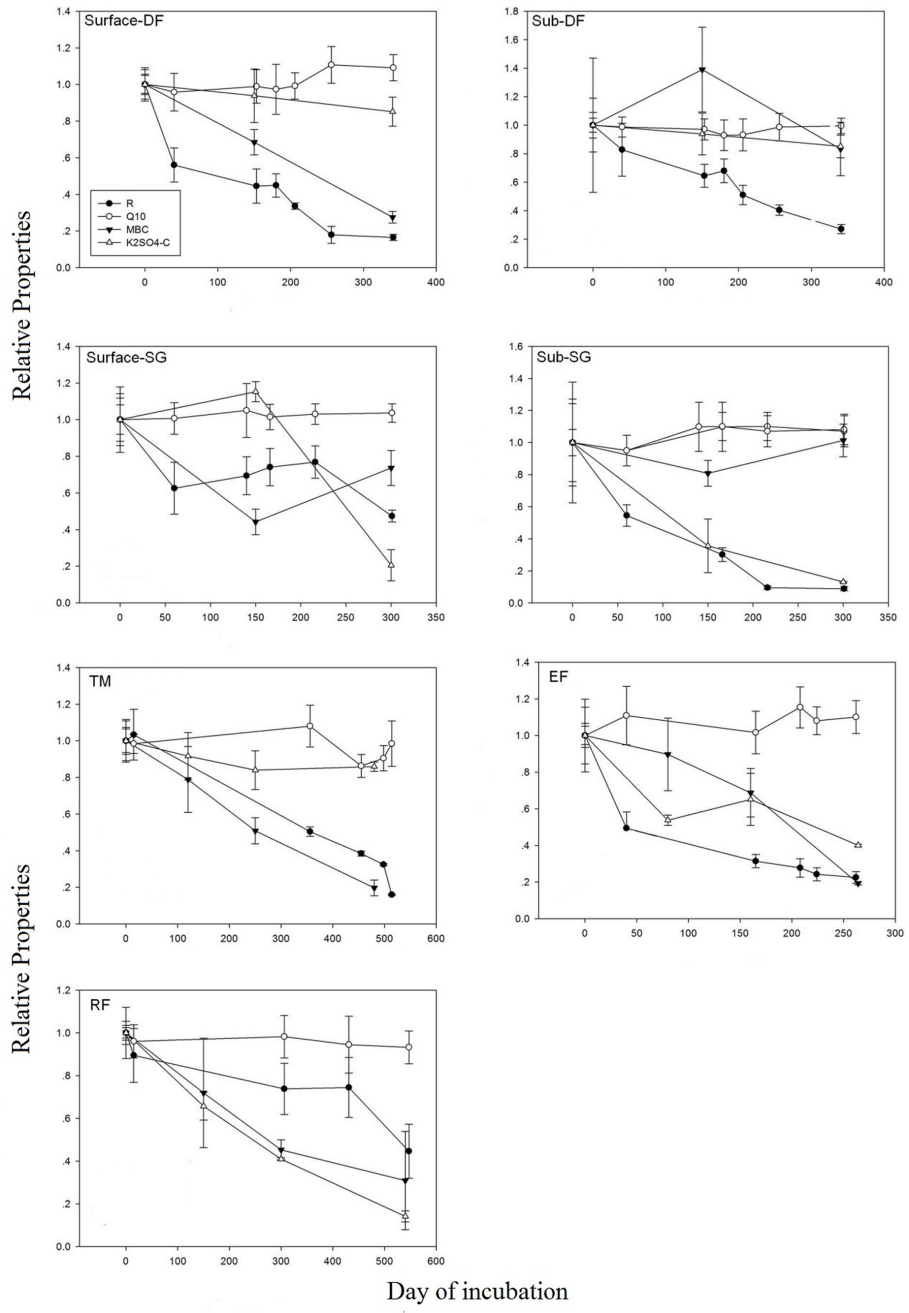
Variation in SOC decomposition rates, *Q*_*10*_ values and soil carbon pools with increasing incubation time. **Values are average of four replications and normalized by initial values**. (DF: deciduous forest; TM: temperate meadow; RF: tropical rainforest; EF: evergreen forest; SG: semi-arid grassland).

Fig 3 shows that respiration rates, MBC and K_2_SO_4_-C concentrations declined significantly along with incubation. Over the whole incubation period, 5 to 7 rounds of *Q*_10_ calculation were obtained for different soil samples. The *Q*_10_ values of soil respiration did not change significantly or systematically with incubation time. In samples from the surface soil of deciduous forest, semi-arid grassland and evergreen forest and subsoil of semi-arid grassland, temperature sensitivity increased slightly with incubation time, the ratio of last *Q*_10_ value to the initial value were 1.09, 1.04, 1.10 and 1.08, respectively, and the temperature sensitivity of samples from the subsurface soil of semi-arid grassland and the surface soil of temperate meadow and tropical rainforest decreased to 0.995, 0.985 and 0.934 of the initial *Q*_10_ value, respectively, but none of the variations was significant (p > 0.05).

### Effect of soil depth on estimated Q_10_ value

**Fig 4:**
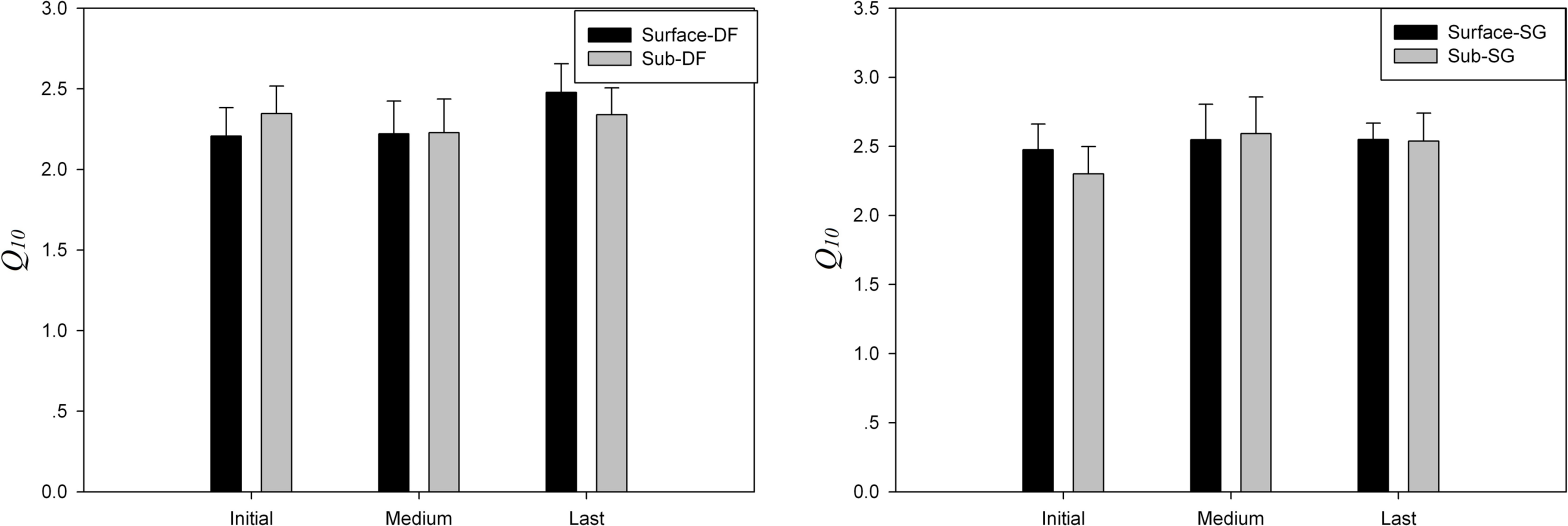
Comparison of the *Q*_*10*_ value of samples from the same site but different layer over the incubation. **No significant difference was found in both semi–arid grassland samples and the deciduous forest samples.** (DF: deciduous forest; SG: semi-arid grassland).

According to changes in respiration rate, the whole incubation period was divided into three phases: the initial phase (0–60 days), the second phase (61–210 days) and the last phase (after 210 days). In each phase, *t—*test was used to compare the average *Q*_10_ values of the surface soil with that of subsurface soil of each site. In the initial and second phases, the average *Q*_10_ value of surface soil samples from deciduous forest were higher than that of subsurface soil from the same site, while the *Q*_10_ value of surface soil was lower than in the last phase. The average *Q*_10_ value of surface sample from semi-arid grassland was higher than that of subsurface sample in the initial phase, but approximately equal to in the second and last phases (Fig 4). Statistically, there were no significantly differences (p > 0.05) between *Q*_10_ values of different layer samples from the same site at each incubation stages.

### Effects of ecosystem types on Q_10_ value

**Fig 5:**
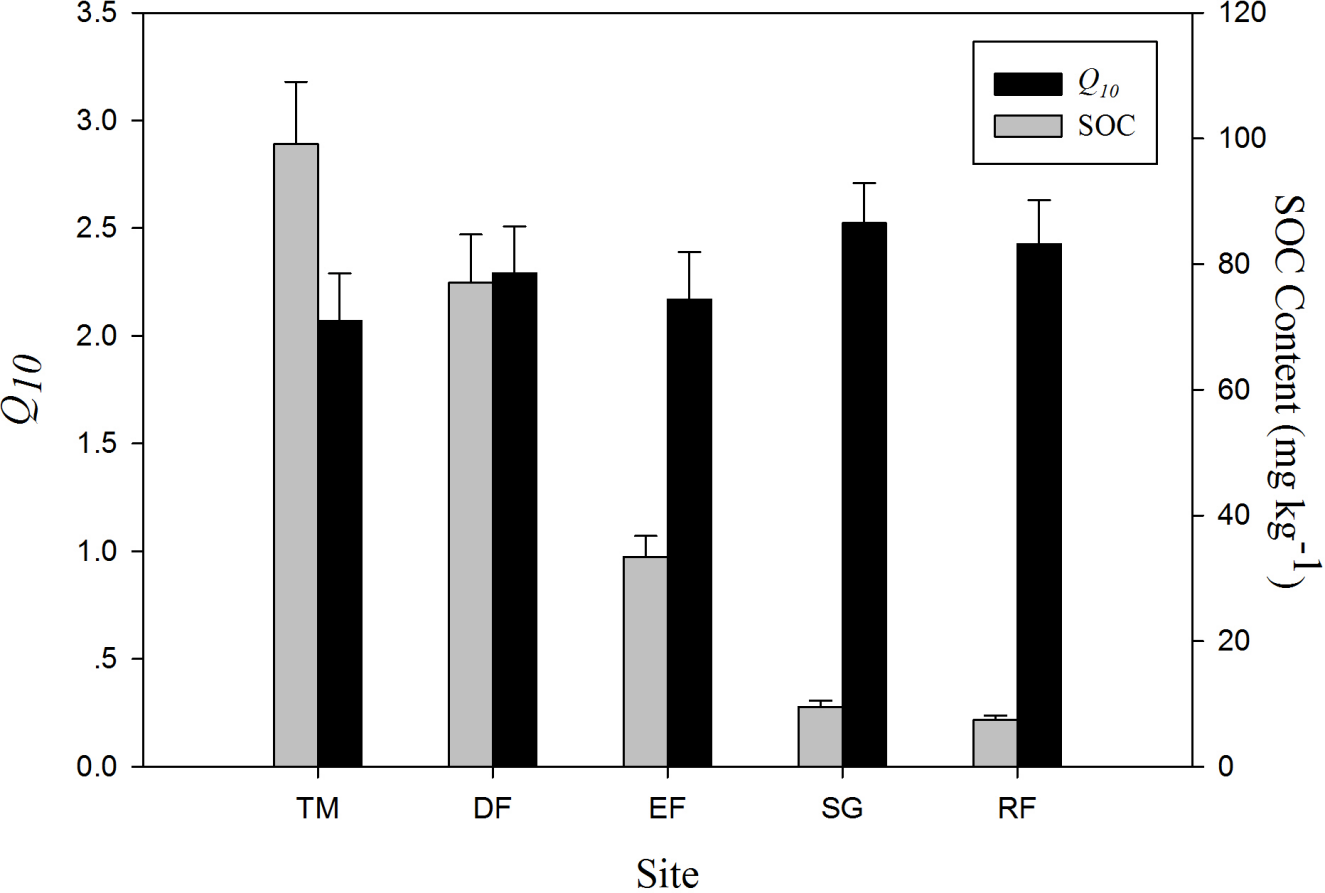
The *Q*_*10*_ value of each ecosystem coupled with SOC content. ***Q***_***10***_ **value is the average *Q***_***10***_ **value over the whole incubation of each surface soil.** (DF: deciduous forest; TM: temperate meadow; RF: tropical rainforest; EF: evergreen forest; SG: semi-arid grassland).

As the *Q*_*10*_ value did not vary significantly during the incubation of each sample, we used the average *Q*_*10*_ value over the incubation of each surface soil sample to stand for the *Q*_*10*_ value of each ecosystem. It was found that the relative high *Q*_*10*_ values appeared in both highest-latitude and lowest-latitude locations, one of which was grass land and the other was forest. Coupled average *Q*_*10*_ values with SOC content, it was showed that high SOC content tended to low *Q*_*10*_ value (Fig 5).

## Discussions

In our study, soil respiration was initially higher in most of surface samples than that in subsurface, indicating a better organic C quality in organic layers, which can proved by the higher contents of SOC, MBC and total N [8]. Soil respiration decreased rapidly at the early stage of incubation, but the decrement slowed down at the late stage, suggesting that labile organic matter may be depleted quickly along with incubation, such phenomenon has also been observed in other studies [16,24,25]. The respiration rates of both surface and subsurface samples from tropical rainforest were relatively low at the beginning of incubation and decreased slightly with the progress of incubation, implying that these samples contained more relatively resistant C pools than other sites.

For an individual sampling site, it is commonly accepted that the soil from deeper layer is “older” or more resistant to decomposition than upper soil layer [8,16]. This study revealed that no matter how different of SOC composition caused by soil depth or incubation process, there was no systematic changes in estimated *Q*_10_ values of soil respiration/SOC decomposition. Based on the method suggested by previous study [8] to estimate the relative contribution from labile and resistant C pool to the apparent *Q*_10_ value of SOC decomposition, our results confirmed that the temperature sensitivity of labile carbon component decomposition was approximately equal to that of resistant component, in agreement with other studies [8,10,26].

It was suggested that the decomposition of low quality SOC involves higher activation energies and therefore would be more temperature sensitive than high quality SOC [27]. However, there are many other factors influencing the decomposition progress which confounds the temperature response of decomposition. In long-term laboratory incubation studies, the substrate content may constrain the decomposition rate at late stage of incubation, when more resistant component is decomposed, and the low substrate availability may lead to a canceling effect which would suppress the intrinsic temperature dependency of SOC decomposition. The addition of readily available substrates significantly increased *Q*_10_ values because substrate saturation eliminated the canceling effect on estimated *Q*_10_ values [28]. Other processes may cancel or enhance the effects of each other [29], leading to contradictory results. Thus, the apparent *Q*_*10*_ value in situ cannot be simply described as the activation energy of Arrhenius equation.

**Fig 6:**
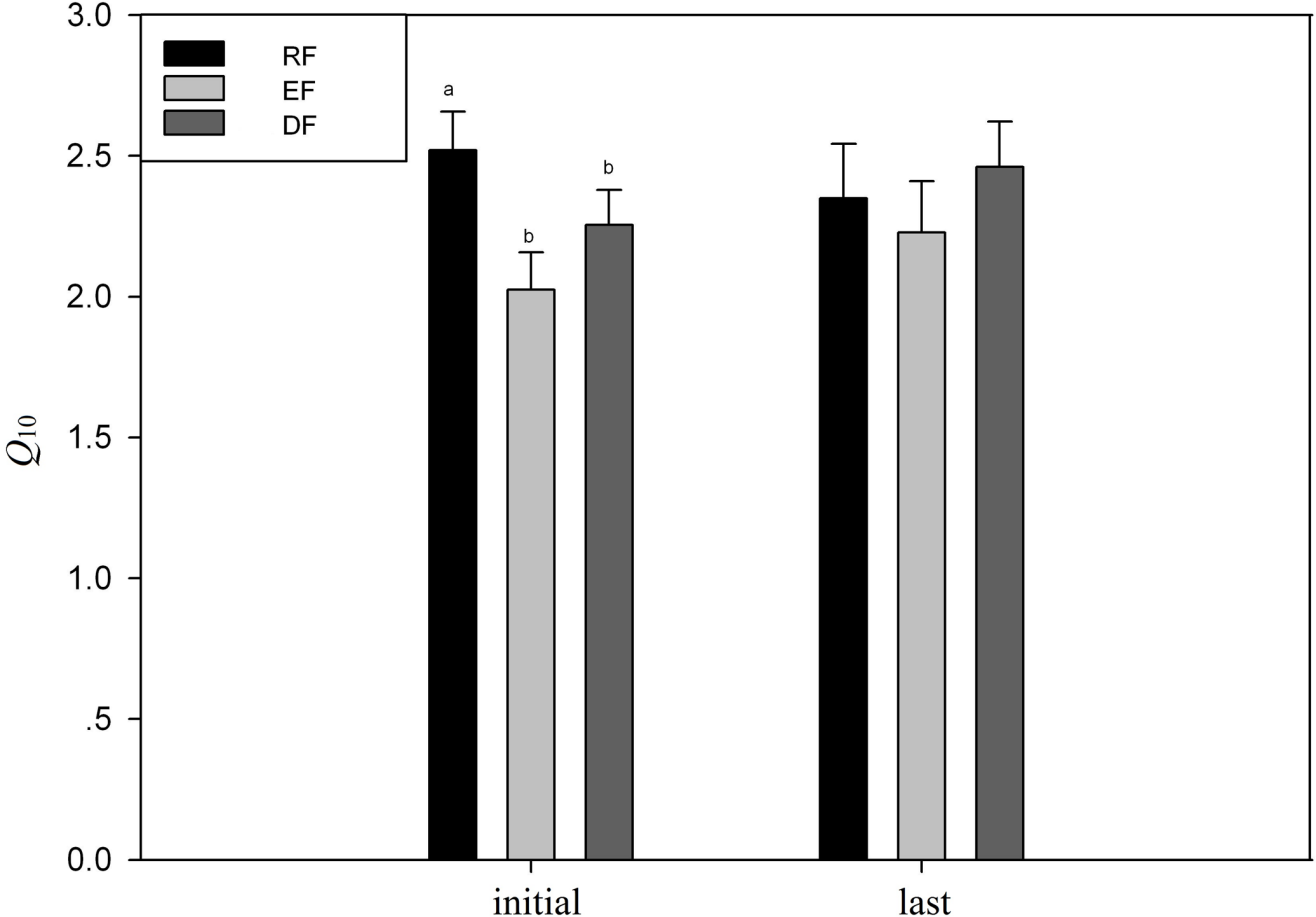
Comparison of the initial and last *Q*_10_ value of three forest samples. (DF: deciduous forest; RF: tropical rainforest; EF: evergreen forest).

Comparing the *Q*_10_ derived from different sites, it was found that surface soil samples from three different forests have similar final *Q*_10_s but different initial *Q*_10_s (Fig 6). At the beginning of incubation, the *Q*_10_ of deciduous forest, evergreen forest and rain forest were 2.25 ± 0.12, 2.02 ± 0.13, 2.52 ± 0.14, respectively, and the *Q*_10_ value of rain forest was significantly higher than other two sites. Commonly, more recalcitrant component is expected in low latitude soils due to high SOC turnover rate caused by high MAT, comparing with the soils in higher latitude [30], which was verified by the lower contents of SOC, MBC, K_2_SO_4_-C and total N. If the recalcitrant C pool is more temperature sensitive than labile pool, a general pattern of *Q*_10_ in deciduous forest < evergreen forest < rain forest is expected. Despite that *Q*_10_ value in rain forest is the greatest in all three soils, the *Q*_10_ values in all three soils did not clearly support or reject the point of view that the recalcitrant C is more temperature sensitive that labile C. At the end of incubation, *Q*_10_ values for the three soils were 2.46 ± 0.16, 2.23 ± 0.18, 2.35 ± 0.19, respectively, with no statistical significance, which implicated the different SOC components would have similar temperature sensitivity in some condition. One possible explanation to the similar *Q*_10_ values among different forest soils is that soil microbial communities had been changed during incubation, which has been observed in other studies [18,23,31]. The temperature sensitivity of substrate-induced respiration has been reported to be significantly related to microbial communities [32]. Under the same incubation environment, the microbial communities’ structure might tend to be similar among the three soils, which led the similar temperature sensitivity of SOC decomposition to different SOC components.

Current debate about the temperature sensitivity of different SOC fractions is at least partly because of the various methods in soil incubation and estimating temperature sensitivity. Details of experiment setting and calculating method might lead to different conclusions. It was pointed out that the assumption of fixed residence times would lead to a bias estimate on *Q*_*10*_ value [33]. Similarly, the presumption of same reference decay rate for all pools in Knorr’ study [11] necessarily leads to the conclusion that the resistant pool is more temperature sensitive than the labile pool [34]. Through re-analyzing the same data of SOC turnover in Vanhala’s study [13] with very similar but different way, and draw a different conclusion that old SOC did not have a higher *Q*_10_ value than young SOC [9]. In an incubation experiment [14], a certain recalcitrant carbon component were respired at day about 80 to 105 under 35°C, while the same component were respired at day about 210 to 260 under 25°C, however, along with the progress of incubation, soil respiration rate declined significantly [8,16], at least partly due to the decline of soil microbial, thus, estimating *Q*_10_ by the ratio of incubation time for the same component under different temperature would result in an overestimated *Q*_10_ value of SOC decomposition, and therefore overestimating the temperature sensitivity of the decomposition of recalcitrant carbon. Some research showed that *Q*_10_ values of acid hydrolysis residues were lower than particulate organic matters (labile fractions in their experiment) but the *Q*_10_ values of 18h hydrolysis residues were higher than 1 and 6 h hydrolysis residues, which implied two contrast results [15].

**Fig 7:**
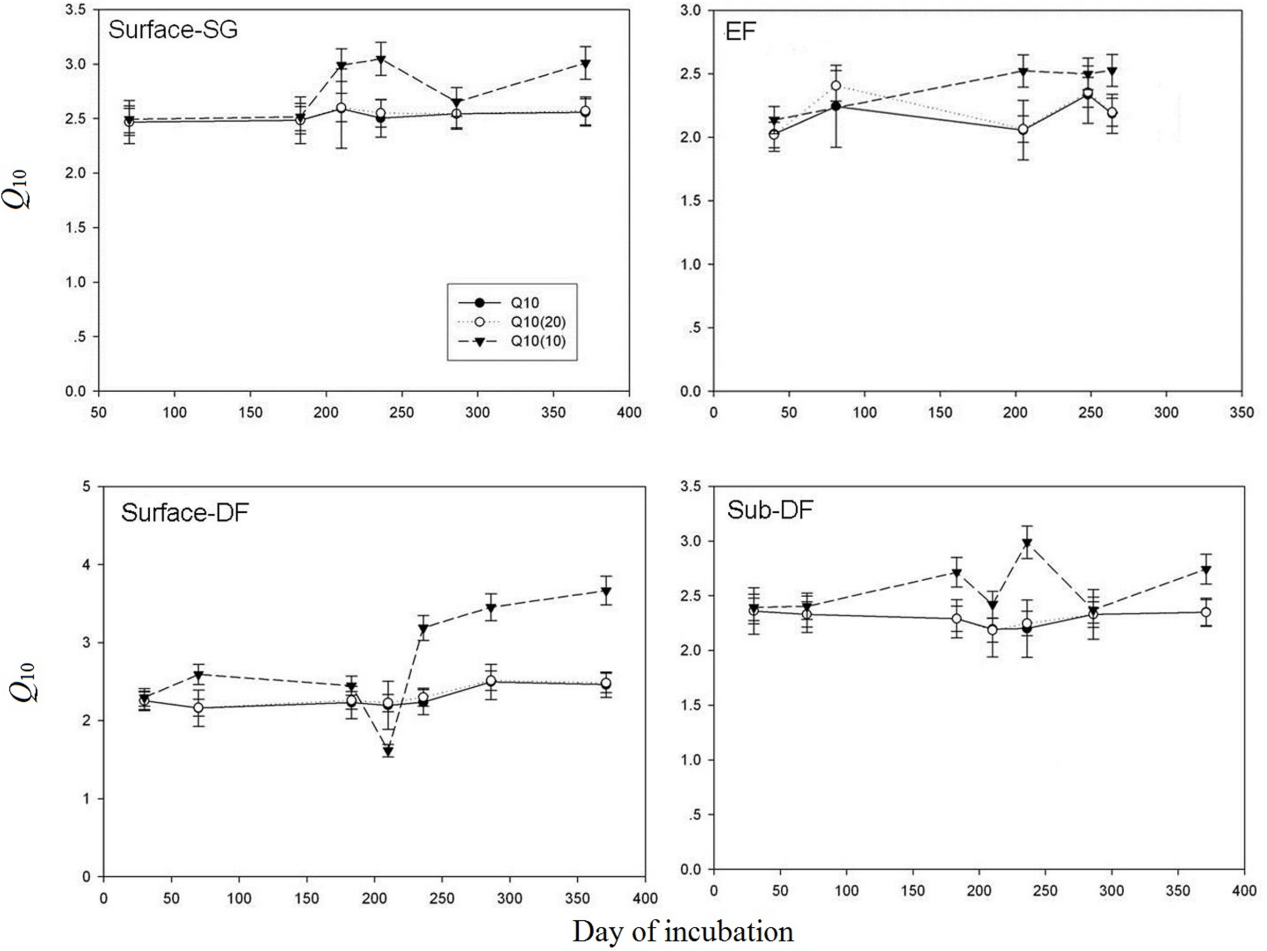
The variation of *Q*_10_ values calculated with two different models along with incubation time. *Q*_10_(20) was the *Q*_10_ value calculated at 20 ^o^C and *Q*_10_(10) was the Q_10_ value calculated at 10 ^o^C by the O’connel model, while *Q*_10_ was the *Q*_10_ value calculated by first–ordered exponential model. (DF: deciduous forest; EF: evergreen forest; SG: semi-arid grassland).

In our study, we calculated the variation of *Q*_10_ values with increasing incubation time by O’connel model and first–ordered exponential model, which showed some different results. For example, the *Q*_10_ values calculated by O’connel model at 20°C nearly keep the same with the *Q*_10_ values calculated by First–ordered exponential model, and remained unchanged during the incubation, while the *Q*_10_ values calculated by O’connel model at 10°C show an increasing trend with increasing incubation time. (Fig 7). The *Q*_10_ values derived from commonly used respiration models, except from the First–ordered exponential model, is changed with temperature, which would lead different results while comparing the *Q*_10_ values of different SOC fractions under different calculating temperature as some research showed [16].It seems that we need a more precise parameter instead of *Q*_10_ value and more valid experiment methods to figure out whether and how the different SOC fractions respond to temperature.

As the decomposition of SOC is the progress of converting SOC into CO_2_ or CH_4_, and mainly microbiologically mediated [35], the temperature sensitivity of SOC decomposition should be the response of microbial activity to temperature change. That is, the *Q*_10_ values of organic matter decomposition should be mainly affected by micro-organisms rather than other factors. It was thought that the different incubation temperature caused changes in the composition of microbial flora, resulting in different temperature sensitivities during the incubation [18]. Other study pointed that the supply of substrate and the affinity of enzyme and substrate would both affect the temperature sensitivity of decomposition, which meant the microbial characteristics would affect the *Q*_10_ value [36]. If we assume that the temperature sensitivity of SOC decomposition is mainly due to the response of microbes to temperature change, results of this study that the temperature sensitivity of SOC decomposition is not related to labile and recalcitrant carbon is explainable.

## Conclusion

Along with the progress of a long-term incubation of five soils developed under different climate regions and ecosystems, soil heterotrophic respiration rates and contents of microbial biomass carbon and K_2_SO_4_ extractable carbon decreased significantly, suggesting a decline in SOC quality. However, the *Q*_10_ value of soil heterotrophic respiration/SOC decomposition did not show a significantly systematic change in respect to the variation in SOC components caused by incubation, soil sampling at different profile depth, different climate conditions and ecosystem types. In this study, labile C component and recalcitrant C component from various ecosystem types response similar to temperature change, suggesting that temperature sensitivity may not related to SOC quality. Present experimental methods or existing models resulted in large uncertainties in estimated *Q*_10_ value of soil respiration or SOC decomposition, hence, limiting our ability to quantitatively ascribe estimated temperature sensitivity to SOC components.

## Supporting information

S1 Table Respiration rates of surface soil samples. (DOCX)

S2 Table Respiration rates of sub-surface soil samples. (DOCX)
