# Supplementary material for: The temperature sensitivity of soil organic carbon decomposition is not related to labile and recalcitrant carbon
Source: PLoS One. 2017 Nov 2;12(11):e0186675. doi: 10.1371/journal.pone.0186675 (PMC5667802; doi:10.1371/journal.pone.0186675)
Supplement: S1 Table — (DOCX) [file pone.0186675.s001.docx]

**S1 Table. Respiration rates of surface soil samples （Average±SD , unit : umol CO_2_﹒g^-1^soil﹒h^-1^)**

| Sample sites ID | Incubation Time  (Days) | Incubation temperature(^o^C) | | | | | |
| --- | --- | --- | --- | --- | --- | --- | --- |
| RF |  | 12 | 16 | 20 | 24 | 28 | 32 |
|  | 0 | 0.025+0.003 | 0.034+0.003 | 0.052+0.005 | 0.065+0.002 | 0.103+0.008 | 0.149+0.004 |
|  | 15 | 0.025+0.006 | 0.032+0.005 | 0.047+0.006 | 0.069+0.008 | 0.099+0.011 | 0.138+0.01 |
|  | 306 | 0.016+0.002 | 0.028+0.005 | 0.04+0.005 | 0.062+0.006 | 0.101+0.008 | 0.117+0.008 |
|  | 431 | 0.017+0.005 | 0.022+0.004 | 0.028+0.005 | 0.05+0.006 | 0.066+0.009 | 0.091+0.016 |
|  | 547 | 0.011+0.002 | 0.014+0.002 | 0.023+0.006 | 0.032+0.003 | 0.044+0.003 | 0.062+0.004 |
| EF | 0 | 0.111+0.019 | 0.148+0.016 | 0.215+0.029 | 0.277+0.033 | 0.354+0.038 | 0.477+0.041 |
|  | 41 | 0.047+0.019 | 0.09+0.006 | 0.106+0.017 | 0.296+0.037 | 0.333+0.025 | 0.346+0.033 |
|  | 165 | 0.039+0.009 | 0.034+0.01 | 0.069+0.009 | 0.108+0.017 | 0.105+0.006 | 0.163+0.029 |
|  | 208 | 0.028+0.004 | 0.039+0.006 | 0.06+0.009 | 0.086+0.01 | 0.111+0.013 | 0.161+0.025 |
|  | 224 | 0.027+0.004 | 0.035+0.006 | 0.052+0.007 | 0.074+0.008 | 0.098+0.01 | 0.131+0.013 |
|  | 262 | 0.024+0.004 | 0.034+0.005 | 0.049+0.006 | 0.068+0.008 | 0.089+0.013 | 0.126+0.014 |
| DF | 0 | 0.234+0.05 | 0.29+0.039 | 0.444+0.033 | 0.613+0.056 | 0.824+0.035 | 1.152+0.051 |
|  | 40 | 0.125+0.027 | 0.179+0.045 | 0.249+0.041 | 0.375+0.043 | 0.455+0.069 | 0.657+0.036 |
|  | 153 | 0.087+0.023 | 0.138+0.032 | 0.198+0.036 | 0.262+0.039 | 0.359+0.036 | 0.493+0.046 |
|  | 180 | 0.143+0.011 | 0.127+0.027 | 0.208+0.021 | 0.189+0.043 | 0.356+0.061 | 0.485+0.062 |
|  | 206 | - | 0.082+0.021 | 0.149+0.007 | 0.235+0.086 | 0.277+0.015 | 0.357+0.021 |
|  | 256 | 0.025+0.008 | 0.051+0.011 | 0.08+0.018 | 0.11+0.018 | 0.163+0.018 | 0.221+0.004 |
|  | 341 | 0.026+0.005 | 0.049+0.007 | 0.073+0.006 | 0.11+0.013 | 0.16+0.01 | 0.21+0.008 |
| TM | 0 | 0.183+0.032 | 0.199+0.042 | 0.331+0.019 | 0.486+0.018 | 0.69+0.029 | 0.804+0.027 |
|  | 15 | 0.183+0.031 | 0.236+0.033 | 0.342+0.04 | 0.465+0.026 | 0.605+0.036 | 0.817+0.041 |
|  | 356 | 0.17+0.018 | 0.228+0.036 | 0.334+0.074 | 0.484+0.062 | 0.654+0.093 | 0.911+0.098 |
|  | 455 | 0.151+0.02 | 0.2+0.026 | 0.255+0.039 | 0.33+0.035 | 0.408+0.047 | 0.53+0.042 |
|  | 498 | 0.114+0.017 | 0.155+0.021 | 0.215+0.015 | 0.269+0.025 | 0.34+0.036 | 0.453+0.05 |
|  | 514 | - | 0.057+0.018 | 0.101+0.01 | 0.14+0.012 | 0.183+0.027 | 0.242+0.043 |
| SG | 0 | 0.046+0.008 | 0.065+0.008 | 0.1+0.016 | 0.151+0.019 | 0.193+0.015 | 0.294+0.026 |
|  | 60 | 0.029+0.005 | 0.042+0.007 | 0.063+0.012 | 0.091+0.013 | 0.126+0.015 | 0.184+0.015 |
|  | 140 | 0.018+0.009 | 0.042+0.014 | 0.069+0.009 | 0.078+0.012 | 0.135+0.027 | 0.184+0.023 |
|  | 166 | - | 0.029+0.001 | 0.053+0.006 | 0.074+0.009 | 0.103+0.005 | 0.149+0.007 |
|  | 216 | 0.024+0.003 | 0.037+0.003 | 0.048+0.003 | 0.077+0.008 | 0.11+0.009 | 0.158+0.008 |
|  | 301 | 0.023+0.002 | 0.034+0.004 | 0.047+0.003 | 0.074+0.005 | 0.111+0.007 | 0.153+0.006 |
