# Supplementary material for: The temperature sensitivity of soil organic carbon decomposition is not related to labile and recalcitrant carbon
Source: PLoS One. 2017 Nov 2;12(11):e0186675. doi: 10.1371/journal.pone.0186675 (PMC5667802; doi:10.1371/journal.pone.0186675)
Supplement: S2 Table — (DOCX) [file pone.0186675.s002.docx]

**S2 Table. Respiration rates of sub-surface soil samples （Average±SD , unit : umol CO_2_﹒g^-1^soil﹒h^-1^)**

| Sample sites ID | Incubation Time  (Days) | Incubation temperature(^o^C) | | | | | |
| --- | --- | --- | --- | --- | --- | --- | --- |
| DF |  | 12 | 16 | 20 | 24 | 28 | 32 |
|  | 0 | 0.034+0.008 | 0.051+0.008 | 0.074+0.012 | 0.109+0.013 | 0.136+0.017 | 0.205+0.019 |
|  | 40 | 0.029+0.005 | 0.044+0.007 | 0.061+0.012 | 0.09+0.008 | 0.118+0.01 | 0.17+0.013 |
|  | 153 | 0.023+0.003 | 0.031+0.006 | 0.048+0.005 | 0.067+0.005 | 0.095+0.008 | 0.126+0.014 |
|  | 180 | 0.014+0.004 | 0.032+0.004 | 0.05+0.005 | 0.062+0.002 | 0.068+0.005 | 0.112+0.013 |
|  | 206 | - | 0.014+0.003 | 0.033+0.003 | 0.039+0.005 | 0.05+0.003 | 0.071+0.009 |
|  | 256 | 0.01+0.001 | 0.014+0.002 | 0.022+0.002 | 0.03+0.002 | 0.039+0.005 | 0.058+0.008 |
|  | 341 | 0.008+0.001 | 0.012+0.001 | 0.02+0.002 | 0.027+0.003 | 0.037+0.002 | 0.052+0.003 |
| SG | 0 | 0.036+0.004 | 0.052+0.014 | 0.078+0.017 | 0.11+0.009 | 0.15+0.014 | 0.213+0.018 |
|  | 60 | 0.02+0.003 | 0.028+0.006 | 0.043+0.005 | 0.059+0.012 | 0.079+0.007 | 0.108+0.013 |
|  | 140 | 0.007+0.001 | 0.014+0.001 | 0.025+0.003 | 0.047+0.002 | 0.057+0.004 | 0.084+0.012 |
|  | 166 |  | 0.016+0.002 | 0.024+0.003 | 0.031+0.002 | 0.044+0.002 | 0.069+0.005 |
|  | 216 | 0.003+0 | 0.005+0.001 | 0.007+0.001 | 0.011+0.002 | 0.015+0.001 | 0.022+0.002 |
|  | 301 | 0.003+0 | 0.005+0.001 | 0.006+0.001 | 0.01+0.001 | 0.015+0.001 | 0.02+0.003 |
